# Editorial: Genomic and epigenomic applications in animal and veterinary sciences

**DOI:** 10.3389/fvets.2023.1167079

**Published:** 2023-03-20

**Authors:** Abdul Rasheed Baloch, Jean Magloire Feugang, Nélida Rodríguez-Osorio

**Affiliations:** ^1^Panjwani Center for Molecular Medicine and Drug Research, International Center for Chemical and Biological Sciences, University of Karachi, Karachi, Pakistan; ^2^Department of Animal and Dairy Sciences, Mississippi State University, Starkville, MS, United States; ^3^Unidad de Genómica y Bioinformática, Departamento de Ciencias Biológicas, Universidad de la República, Salto, Uruguay

**Keywords:** animal genomics, animal epigenomics, domestic animals and wildlife, veterinary research, animal sciences

In the last two decades, all biological and health disciplines, from virology to ecology, have been revolutionized by genomics with the advances in sequencing technologies and bioinformatics software development. Animal and veterinary sciences have not been an exception; the impact of genomics, transcriptomics, proteomics, and other “omic” branches on animal selection, breeding, nutrition, and health is evident.

Among the plethora of genomic and epigenomic papers published each year, the proportion of publications involving non-model, domestic, or wild animals is still low. However, the availability of annotated genomes for most domestic and economically relevant species (Table 1 collects genome assembly information for some domestic animals) has made it possible for animal science and veterinary researchers to turn to cutting-edge genomic strategies to better identify genetic markers associated with productive traits, characterize pathologic agents, associate transcriptomic information with physiological responses, and evaluate the effect of epigenetic modifications on gene expression. Even multi-omic data integration approaches are being applied to animal research (11).

**Table 1:** Assembly information for important domestic species genomes, organized chronologically according to the first assembly release.

**Species**	**First assembly**		**Current reference assembly**		
	**Year**	**Submitter**	**Identifier**	**Accession**	**Last update**
Dog, *Canis lupus familiaris*	2003	Kirkness et al. (1)	Dog10K_Boxer_Tasha	GCA_000002285.4	2020
Chicken, *Gallus gallus*	2004	International Chicken Genome Sequencing Consortium (2)	GRCg6a	GCA_000002315.5	2018
Cat, *Felis catus*	2007	Pontius et al. (3)	F.catus_Fca126_mat1.0	GCA_018350175.1	2021
Horse, *Equus caballus*	2007	Wade et al. (4)	EquCab3.0	GCA_002863925.1	2018
Cattle, *Bos taurus*	2009	Bovine Genome Sequencing and Analysis Consortium (5)	ARS-UCD1.3	GCA_002263795.3	2018
Rabbit, *Oryctolagus cuniculus*	2009	Unpublished	UM_NZW_1.0	GCA_009806435.2	2021
Pig, *Sus scrofa*	2009	Archibald et al. (6)	Sscrofa11.1	GCA_000003025.6	2017
Sheep, *Ovis aries*	2010	Archibald et al. (7)	ARS-UI_Ramb_v2.0	GCA_016772045.1	2021
Turkey, *Meleagris gallopavo*	2010	Dalloul et al. (8)	Turkey_5.1	GCA_000146605.4	2019
Tilapia, *Oreochromis niloticus*	2012	Brawand et al. (9)	O_niloticus_UMD_NMBU	GCA_001858045.3	2018
Buffalo, *Bubalus bubalis*	2013	Unpublished	NDDB_SH_1	GCA_019923935.1	2021
Goat, *Capra hircus*	2013	Bickhart et al., Unpublished	ARS1.2	GCA_001704415.2	2016
Duck, *Anas platyrhynchos*	2013	Huang et al. (10)	ZJU1.0	GCA_015476345.1	2020

This Research Topic, on the application of genomic and epigenomic tools in animal and veterinary sciences, aimed at showcasing research that included genomic and epigenomic tools to solve questions relating to animal and veterinary science. The result is a set of nine original research papers and two review articles that explore a diverse array of applications of genomics to animal health, reproduction, production, and response to stress and pathogens in cattle, yak, Chinese forest musk deer, pig, rat, chicken, and carp.

Two papers focused on measuring microRNA expression in domestic animals: Wang H. et al. evaluated the role of miR-22 in Suhuai pig skeletal muscle and identified a SNP upstream of the miR-22 precursor sequence that correlates with pork color. The target of this microRNA is ELOVL6, an elongase that catalyzes *de novo* synthesis of fatty acids, previously linked with the regulation of muscle fiber type conversion, and that appears to be over-expressed in white muscle. Their findings provide a basis for future research on the molecular markers of pork color. The paper by Veshkini et al. studied circulating miRNAs in dairy cows to assess their roles in the metabolic adaptations the cow goes through before and after calving. According to their results, calving significantly affects miRNA expression, disturbing signaling pathways related to energy, metabolism, and immunity.

Three additional papers used genomic tools for association and gene expression studies. Khan et al. genotyped *TRAPPC9* and *CD4* polymorphisms in Chinese Holstein cows and studied their association with milk production and mastitis resistance. They associated the identified SNPs with milk production, protein content, somatic cell count, somatic cell score, and the expression of interleukin 6 (IL-6) and interferon-gamma (IFN-γ), suggesting that polymorphisms in these genes could be useful markers for milk production and mastitis resistance in dairy cattle. The pioneering work by Chen et al. employed Nanopore long-read RNA-Seq to evaluate the genome-wide allelic differential expression in the lung and liver of domesticated cattle-yak hybrids (known as “yattle”). The authors found that genes related to hypoxia adaptation and immune response were predominantly expressed from the yak alleles. In contrast, lipid metabolism and endocrine secretion genes were expressed from the cattle alleles. This analysis of the differential contribution of parental alleles in hybrid animals could enhance our understanding of the genetic basis of hybrid vigor during crossbreeding. Thirdly, a weighted gene co-expression network analysis (WGCNA) was applied by Wang X. et al. to identify key genes involved in the regulation of subcutaneous adipose tissue. They identified 15 gene co-expression modules and selected 3, according to the correlation between modules and phenotype, from which eight hub lipid metabolism genes were identified. Their expression levels were measured in the heart, liver, spleen, lung, kidney, muscle, and adipose tissue. The results provide a theoretical basis for studying beef quality by identifying hub genes that regulate lipid metabolism.

A couple of papers in this Research Topic studied gene expression in brain tissue. Baker et al. explore the pattern of DNA methylation and gene expression in amygdala tissue from Brahman cows exposed to prenatal stress. Although they only found differential methylation in a few individual CpG sites and differentially expressed in two genes, this is one of the first studies on the impact of prenatal stress on cattle brain DNA methylation and transcriptomic profiles. The second paper by Gao et al. measured the protective effects of taurine in rats against the negative effect of formaldehyde, benzene, toluene, and xylene, which are common indoor volatile organic compounds (VOC) and associated them with intellectual and cognitive impairment in children. Taurine protected rats against VOC-induced cognitive-behavioral damage and restored their learning and memory. Therefore, suggesting that it could be a potential treatment for a cognitive behavioral disorder.

Given the association between bacterial infection and pneumonia and the threat, it poses to the endangered Chinese forest musk deer (*Moschus berezovskii*), Tang et al. relied on ITRAQ-based quantitative proteomics to understand pneumonia pathogenesis in this species. Since the forest musk deer genome is poorly annotated, the researchers used the bovine genome to identify the proteins and found a clear dysregulation of proteins involved in bacterial infection and immunity, in deer suffering from pneumonia. These results shed light on the molecular mechanisms, and pathways underlying pneumonia pathogenesis.

The paper by Xie et al. is one of the first attempts at optimizing common carp (*Cyprinus carpio*) germ cell culture, which could open new opportunities for the application of surrogate production, a biotechnological strategy that could be valuable in common carp breeding, the restoration and development of lines, and the conservation of genetic resources.

Finally, this Research Topic included two review papers: first, Gul et al. reviewed the recent genetic basis of poultry resistance against microbial pathogens and genomic modifications that increase resistance against pathogens in chickens. Understanding disease resistance genetics would enable the identification of resistance markers and the development of disease resistance breeds, which could reduce the dependency on vaccination and prophylactic antibiotics in the poultry industry. The last paper by Guo et al.'s team reviews the methods and advances in cell immortalization in livestock and poultry. Immortalized cell lines provide a reliable tool for biological research and stable infinite cell lines should guarantee proliferation, while maintaining normal cell function.

## Author contributions

NR-O initiated the Research Topic and drafted the manuscript. AB, JF, and NR-O co-edited the Research Topic and participated in the editorial process. All authors revised and approved the manuscript.

## References

[1] Kirkness EF Bafna V Halpern AL Levy S Remington K Rusch DB. The dog genome: Survey sequencing and comparative analysis. Science. (2003) 301:1898–903. 10.1126/science.1086432 14512627

[2] International Chicken Genome Sequencing Consortium. Sequence and comparative analysis of the chicken genome provide unique perspectives on vertebrate evolution. Nature. (2004) 432:695–716. 10.1038/nature03154 15592404

[3] Pontius JU Mullikin JC Smith DR Team AS Lindblad-Toh K Gnerre S. Initial sequence and comparative analysis of the cat genome. Genome Res. (2007) 17:1675–89. 10.1101/gr.6380007 17975172 PMC2045150

[4] Wade CM Giulotto E Sigurdsson S Zoli M Gnerre S Imsland F. Genome sequence, comparative analysis, and population genetics of the domestic horse. Science. (2009) 326:865–7. 10.1126/science.1178158 19892987 PMC3785132

[5] Bovine Genome Sequencing and Analysis Consortium Elsik CG Tellam RL Worley KC Gibbs RA Muzny DM Weinstock GM. The genome sequence of taurine cattle: A window to ruminant biology and evolution. Science. (2009) 324:522–8. 10.1126/science.1169588 19390049 PMC2943200

[6] Archibald AL Bolund L Churcher C Fredholm M Groenen MA Harlizius B. Pig genome sequence—Analysis and publication strategy. BMC Genomics. (2010) 11:438. 10.1186/1471-2164-11-438 20642822 PMC3017778

[7] Consortium ISG Archibald AL Cockett NE Dalrymple BP Faraut T Kijas JW. The sheep genome reference sequence: A work in progress. Anim Genet. (2010) 41:449–53. 10.1111/j.1365-2052.2010.02100.x 20809919

[8] Dalloul RA Long JA Zimin AV Aslam L Beal K Blomber LA. Multi-platform next-generation sequencing of the domestic turkey (*Meleagris gallopavo*): Genome assembly and analysis. PLoS Biol. (2010) 8:e1000475. 10.1371/journal.pbio.1000475 20838655 PMC2935454

[9] Brawand D Wagner CE Li YI Malinsky M Keller I Fan S. The genomic substrate for adaptive radiation in African cichlid fish. Nature. (2014) 513:375–81. 10.1038/nature13726 25186727 PMC4353498

[10] Huang Y Li Y Burt DW Chen H Zhang Y Qian W. The duck genome and transcriptome provide insight into an avian influenza virus reservoir species. Nat Genet. (2013) 45:776–83. 10.1038/ng.2657 23749191 PMC4003391

[11] Kim DY Kim JM. Multi-omics integration strategies for animal epigenetic studies—A review. Anim Biosci. (2021) 34:1271–82. 10.5713/ab.21.0042 33902167 PMC8255897

